# Remdesivir for pregnancy: A systematic review of antiviral therapy for COVID-19

**DOI:** 10.1016/j.heliyon.2022.e08835

**Published:** 2022-01-31

**Authors:** David Setyo Budi, Nando Reza Pratama, Ifan Ali Wafa, Manesha Putra, Manggala Pasca Wardhana, Citrawati Dyah Kencono Wungu

**Affiliations:** a Faculty of Medicine, Universitas Airlangga, Indonesia; b Division of Maternal-Fetal Medicine, Department of Obstetrics and Gynecology, University of Colorado Anschutz School of Medicine, Aurora, United States; c Maternal-Fetal Medicine, Department of Obstetrics & Gynecology, Dr. Soetomo Hospital, Faculty of Medicine of Universitas Airlangga, Surabaya, Indonesia; d Department of Physiology and Medical Biochemistry, Faculty of Medicine, Universitas Airlangga, Indonesia; e Institute of Tropical Disease, Universitas Airlangga, Indonesia

**Keywords:** COVID-19, SARS-CoV-2, Pregnancy, Remdesivir, Antiviral therapy

## Abstract

**Objective:**

The use of remdesivir for pregnant patients with coronavirus disease 2019 (COVID-19) showed conflicting results in prior studies. We aimed to systematically review its efficacy and safety for this population from the existing literature.

**Methods:**

On July 26, 2021, registries (ClinicalTrials.gov) and databases (MEDLINE, ScienceDirect, Cochrane Library, JSTOR, DOAJ, and medRxiv) were systematically searched for research articles investigating remdesivir use in pregnant people with COVID-19. Clinical outcome, hospitalization duration, laboratory outcome, mortality, and adverse events were investigated.

**Results:**

We obtained 13 observation studies with 113 pregnant people. In these studies, remdesivir improved the clinical condition of pregnant patients with COVID-19, especially those who had a better clinical status at baseline and received earlier remdesivir treatment. Most fetuses were delivered via cesarean section, primarily because of emergency causes. No vertical transmissions were noted. The most reported adverse event was transaminitis, in which 10-day remdesivir treatment yielded more incidence than the 5-day treatment.

**Conclusions:**

In pregnancy, the use of Remdesivir in combination with other COVID-19 treatments is inconclusive but its use should be followed with careful monitoring of adverse reactions and transaminase enzyme levels. Further studies are required to confirm its finding.

## 1 Introduction

The devastating coronavirus disease 2019 (COVID-19) pandemic has caused considerable defiance to the national healthcare systems of most affected countries [1, 2]. As a result, several strategies have been proposed to treat patients with COVID-19; one of them is specific antiviral treatment [3, 4]. Antivirals that deter protease inhibitors and nucleotide or nucleoside analogs that inhibit viral RNA synthesis have been repurposed for COVID-19 treatments. Nucleoside analogs inhibit reverse transcription and are among the most potent antiviral agents available to fight against the SARS-CoV-2 infection; among them is remdesivir [5, 6] (see Table 1A, Table 1B).

**Table 1A:** Characteristics of the included studies.

Reference	Study design	Country	Sample size	Age (Mean ± SD) or median (IQR)		Disease severity	Comorbidities (n)	Intervention	Concurrent therapy
				Patient age (years)	Gestational age (weeks)				
Burwick et al., 2020 [16]	Cohort	United States	67	33 (21–43)	28 (14–39)	Severe to critical	Obesity (11), asthma (9), gestational diabetes (7), chronic hypertension (6), diabetes mellitus (7)	Remdesivir 200 mg loading dose day 1 + 100 mg once daily for days 2–10	Hydroxychloroquine (37%), azithromycin (34%), tocilizumab (1.5%), and lopinavir/ritonavir (1.5%)
Nasrallah et al., 2021 [17]	Cohort	United States	24	32 (16–44)	29 2/7 (6 4/7 to 40 0/7)	Moderate	Obesity (11), asthma (2), hypertension (1)	Remdesivir 200 mg loading dose day 1 + 100 mg once daily for days 2–5	Glucocorticoids, azithromycin, ceftriaxone
Naqvi et al., 2020 [18]	Case Report	United States	1	35	22	Severe	Hypertension, type 2 diabetes mellitus, and asthma	Remdesivir 200 mg loading dose day 1 + 100 mg once daily for days 2–4	Tocilizumab
Maldarelli et al., 2020 [19]	Case Report	United States	1	39	34	Severe	None	Remdesivir 200 mg loading dose day 1 + 100 mg once daily for days 2–10	Hydroxychloroquine
Anderson et al., 2020 [20]	Case Report	United States	1	35	22	Critical	Type 2 diabetes mellitus, asthma, and class III obesity	Remdesivir 200 mg loading dose day 1 + 100 mg once daily for days 2–10	Plasma convalescent, ceftriaxone, azithromycin, and hydroxychloroquine
Jacobson et al., 2020 [21]	Case Report	United States	1	42	26	Severe	None	Remdesivir 200 mg loading dose day 1 + 100 mg once daily for days 2–10	Dexamethasone, convalescent plasma, azithromycin and ceftriaxone
Igbinosa et al., 2020 [22]	Case Series	United States	3	27.3 ± 2.0	28 ± 5.1	Severe	Intrahepatic cholestasis of pregnancy (1)	Remdesivir 200 mg loading dose day 1 + 100 mg once daily for days 2–5	NR
McCoy et al., 2020 [23]	Case Series	United States	5	33.8 ± 6.0	26.4 ± 6.1	Severe	Asthma (2), type 2 diabetes mellitus (1), chronic hypertension (2), obesity (1), chronic kidney disease (1), gestational diabetes (1)	Remdesivir 200 mg loading dose day 1 + 100 mg once daily for days 2–10	Hydroxychloroquine
Saroyo et al., 2021 [24]	Case Series	Indonesia	5	30.2 ± 3.1	32.4 ± 5.6	Moderate to severe	Chronic hypertension (1)	Remdesivir 200 mg loading dose day 1 + 100 mg once daily for days 2–5	NR
Dande et al., 2020 [25]	Case Report	United States	1	39	29	Severe	Rheumatoid arthritis, Sjogren's syndrome	Remdesivir 200 mg loading dose day 1 + 100 mg once daily for days 2–5	Hydroxychloroquine
Singh and Choudhary, 2021 [26]	Case Series	India	2	30.5 ± 2.1	30.5 ± 2.1	Severe	None	10-day course of remdesivir	Dexamethasone, enoxaparin, ceftriaxone, levetiracetam
Chinen et al., 2021 [27]	Case Report	Japan	1	29	34	Critical	None	Remdesivir 200–100 mg/day	Ciclesonide, dexamethasone, heparin sodium, sulbactam/ampicillin, and tocilizumab
Schnettler et al., 2020 [28]	Case Report	United States	1	39	31	Severe	Myotonic dystrophy, bicuspid aortic valve, history of a previous mild cerebrovascular accident	10-day course of remdesivir	Hydroxychloroquine

IQR, interquartile range; NR, not reported; SD, standard deviation.

**Table 1B:** Outcomes of the individual studies.

Reference	Clinical outcome	Hospitalization duration (Mean ± SD) or median (IQR)	Laboratory outcome	Mortality		AE (n)	Pregnancy and neonatal outcome
				Pregnant women	Neonates		
Burwick et al., 2020 [16]	93% of patients recovered within 28 days (96% exhibiting 1 point improvement on the ordinal scale; 93% experiencing 2 points improvement on the ordinal scale).	3 (2–5) days	ALT grade 1 = 10 (16%), grade 2 = 8 (13%), grade 3 = 6 (9%), grade 4 = 0 (0%)	0	1 (spontaneous miscarriage)	- Any AE (22) - Anemia (2) - Deep vein thrombosis (2) - Dysphagia (2) - Unspecified hypertension (2) - Hypoxia (2) - Nausea (2) - Pleural effusion (2) - ARDS (1) - Serious AE (12) - AE leading to discontinuation (7)	Pregnancy outcome: • Among 26 deliveries, 19 (73%) were cesarean and of these, 17 (89%) were emergent. • Most had a high-risk pregnancy due to underlying medical conditions. Neonatal outcome: • No neonatal deaths were reported during the observation period. • One spontaneous miscarriage at 17 gestational weeks occurred in a 32-year-old woman, owing to concurrent bacteremia, tricuspid valve endocarditis, and septic arthritis.
			AST grade 1 = 10 (16%), grade 2 = 12 (19%), grade 3 = 3 (5%), grade 4 = 0 (0%)				
			Creatinine grade 1 = 2 (3%), grade 2 = 5 (8%), grade 3 = 1 (2%), grade 4 = 3 (5%)				
Nasrallah et al., 2021 [17]	On HD7, 100% of patients who received remdesivir <48 h after admission recovered; 0% of patients who started remdesivir >48 h after admission recovered; and 27% of patients treated without remdesivir recovered.	Remdesivir <48 h from admission = 7 days, Remdesivir >48 h from admission = 9–18 days	Laboratory test on admission: Remdesivir <48 h from admission AST (IU/L): 37 (22–90); ALT (IU/L): 30 (14–76); creatinine (mg/dL): 0.6 (0.5–0.7)	0	0	Elevated transaminase (8)	Pregnancy outcome: • Among 27 deliveries, 11 (40.7%) delivered via cesarean section. • Incidental oligohydramnios was seen in 3/24 (12.5%) of women within 5 days of remdesivir treatment. Neonatal outcome: • Apgar score at 5 min was 8–9. • Four infants (14.8%) had fetal growth restriction (1st–7th percentile). • No cases of vertical transmission were reported. • No major histopathologic alterations in the placenta were noted.
			Remdesivir >48 h from admission AST (IU/L): 23 (20–50), ALT (IU/L): 14 (10–38), creatinine (mg/dL): 0.6 (0.5–0.6)				
Naqvi et al., 2020 [18]	On HD9, the patient who received 5-days remdesivir treatment recovered and no longer required oxygen supplementation.	8 days	ALT (units/L) = HD1: 11, HD3: 9, HD5: 12, HD6: 13, HD7: 14, HD8: 14, HD9: 16	0	NA	None	NA
			AST (units/L) = HD1: 21, HD3: 19, HD5: 22, HD6: 21, HD7: 22, HD8: 18, HD9: 16				
			Creatinine (mg/dL) = HD1: 0.50, HD2: 0.57, HD3: 0.58, HD4: 0.53, HD5: 0.53, HD6: 0.53, HD7: 0.53, HD8: 0.53, HD9: 0.54				
Maldarelli et al., 2020 [19]	The patient's supplemental oxygen requirement decreased steadily after receiving 5 doses of remdesivir. On HD9, the patient was discharged after having completed 8 of the 10-day course of remdesivir treatment.	9 days	ALT (units/L) = HD1: 43, HD2: 41, HD3: 37, HD4: 40, HD5: 62, HD6: 62, HD7: 52, HD8: 47	0	0	Transaminitis	After a full recovery from COVID-19, the patient had an uncomplicated spontaneous delivery at term.
			AST (units/L) = HD1: 85, HD2: 78, HD3: 62, HD4: 62, HD5: 83, HD6: 72, HD7: 53, HD8: 46				
Anderson et al., 2020 [20]	The patient was extubated and placed on supplemental oxygen via nasal cannula after receiving 5 doses of remdesivir. On HD14, the patient was planned for discharge following the 10-day remdesivir treatment.	14 days	ALT (units/L) = HD8: 51	0	NA	Transaminitis	NA
			AST (units/L) = HD8: 49				
Jacobson et al., 2020 [21]	Ventilator requirements gradually increased, and oxygen saturation level of 95% or higher could not be maintained after receiving 10-day course of remdesivir.	52 days	NR	0	0	NR	Pregnancy outcome: • Cesarean section was performed at 29 gestational weeks. Neonatal outcome: • Apgar score at 1 and 5 min was 3 and 6, respectively. Neonatal ICU admission was required. • Placental pathology showed few patchy areas, suggesting maternal vascular malperfusion. • SARS-CoV-2 test of the infant on days 3 and 14 were negative.
Igbinosa et al., 2020 [22]	Oxygen supplementation was discontinued in all patients after remdesivir initiation.	7.6 ± 1.5 days	**Case 1** ALT (units/L) = admission: 18, discharge: 432, AST (units/L) = admission: 33, discharge: 457	0	NR	Transaminitis (1)	Uncomplicated spontaneous delivery at 37 weeks occurred in case 1.
			**Case 2** ALT (units/L) = admission: 16, discharge: 14, AST (units/L) = admission: 26, discharge: 16				
			**Case 3** ALT (units/L) = admission: 16, discharge: 19, AST (units/L) = admission: 21, discharge: 28				
McCoy et al., 2020 [23]	Three patients required mechanical ventilation. All 5 patients recovered and were ultimately discharged from the hospital on room air. Two patients completed the 10-day remdesivir treatment, 2 were discharged before completion, and 1 discontinued the treatment because of elevated aminotransferase.	12.2 ± 5.7 days	NR	0	0	Elevated aminotransferase (4)	Pregnancy outcome: • Among 5 patients, 3 underwent cesarean delivery, 1 had spontaneous delivery, and 1 had ongoing delivery. Neonatal outcome: • All infants were in good condition and negative from COVID-19.
Saroyo et al., 2021 [24]	The clinical condition rapidly improved after 5 days of remdesivir treatment and showed shorter period of hospitalization. Naso-oropharyngeal swab was negative shortly after finishing the therapy.	8.0 ± 2.2 days	**Case 1** ALT (units/L) = RD1: 23, RD5: 57, AST (units/L) = RD1: 23, RD5: 21	0	0	None	Pregnancy outcome: • Among 5 patients, 4 had emergency cesarean delivery, and 1 had spontaneous delivery. Neonatal outcome: • Apgar scores of the 5 infants were 7/9, 7/8, 8/9, 9/10, and 8/9, respectively.
			**Case 2** ALT (units/L) = RD1: 23, RD6: NA, AST (units/L) = RD1: 36, RD6: NA				
			**Case 3** ALT (units/L) = RD1: 25, AST (units/L) = RD1: 30				
			**Case 4** ALT (units/L) = RD1: 24, RD4: NA, AST (units/L) = RD1: 25, RD4: NA				
			**Case 5** ALT (units/L) = RD1: NA, RD6: NA, AST (units/L) = RD1: NA, RD6: NA				
Dande et al., 2020 [25]	Steady improvement and decreased oxygen requirement (from 2 L/min on HD1 to 1 L/min on HD4) after 5 days of remdesivir treatment. On HD6, oxygen supplementation was weaned off.	6 days	ALT (units/L) = HD1: 12, HD2: 12, HD3: 10, HD4: 11, HD5: 14, HD6: 22	0	0	None	Pregnancy outcome: • Cesarean delivery was performed at 39 gestational weeks. Neonatal outcome: • Infant did not show any illness and signs of respiratory infection after delivery, thereby not tested for COVID-19.
			AST (units/L) = HD1: 18, HD2: 21, HD3: 21, HD4: 21, HD5: 25, HD6: 34				
			Creatinine (mg/dL) = HD1: 0.45, HD2: 0.44, HD3: 0.36, HD4: 0.38, HD5: 0.36, HD6: 0.40				
Singh and Choudhary, 2021 [26]	The oxygen requirement decreased, and the oxygen saturation level improved after the 10-day remdesivir treatment.	11.5 ± 0.7 days	**Case 1** ALT (units/L) = HD2: 38.4, HD4: 116.2, HD6: 146.3, HD8: 119.5, HD10: 69.2 AST (units/L) = HD2: 23.8, HD4: 128.1, HD6: 146.5, HD8: 143.1, HD10: 48.2 CRP (mg/dL) = HD1: 22.4, HD2: 26.5, HD4: 28, HD6: 12, HD8: 4.10, HD10: 1.8	0	0	Hepatic enzyme increased (2)	Pregnancy outcome: • Preterm spontaneous delivery at 36 gestational weeks was reported in case 1. • Cesarean delivery was performed at 38 gestational weeks because of fetal distress in case 2. Neonatal outcome: • Fetal wellbeing was monitored, and no immediate adverse effect was noted.
			**Case 2** ALT (units/L) = HD1: 143, HD4: 121, HD8: 131, HD10: 115 AST (units/L) = HD1: 164.2, HD4: 137, HD8: 151, HD10: 122 CRP (mg/dL) = HD1: 18.2, HD4: 11.5, HD10: 2.2				
Chinen et al., 2021 [27]	On the 4th day of admission, the respiratory condition rapidly worsened. After cesarean section, the respiratory condition deteriorated, requiring mechanical ventilation.	16 days	ALT (units/L) = HD1: 16, HD4: 17, HD5: 17, HD9: 43	0	0	None	Pregnancy outcome: • Emergency cesarean section was performed at 34 gestational weeks. Neonatal outcome: • Apgar score 8. • The infant' breathing and circulation were stable, and chest X-ray showed no abnormal findings. • SARS-CoV-2 tests were negative at 24 and 60 h postpartum. • No evidence of maternal or fetal vascular malperfusion or acute or chronic inflammatory pathology in the placenta were observed.
			AST (units/L) = HD1: 23, HD4: 29, HD5: 29, HD9: 94				
			Creatinine (mg/dL) = HD1: 0.64, HD4: 0.61, HD5: 0.49, HD9: 0.45				
Schnettler et al., 2020 [28]	Patient experienced rapid clinical decompensation and development of severe COVID-19-related ARDS.	On hospital day 17, patient's condition was improving	NR	0	0	NR	Pregnancy outcome: • Nonemergent cesarean section was performed at 32 gestational weeks because of persistent late decelerations. Neonatal outcome: • The results of SARS-CoV-2 RT-PCR test using amniotic fluid and nasopharyngeal swab from the infant were negative.

Abbreviations: AE, adverse events; ALT, alanine transaminase; ARDS, acute respiratory distress syndrome; AST, aspartate aminotransferase; HD, hospital day; RD, remdesivir day; NA, not available; NR, not reported; RT-PCR, reverse transcription polymerase chain reaction.

Despite no tangible evidence of vertical transmission, pregnancy is considered as a high-risk population during an infectious disease outbreak [7]. The fetus is protected by the complex immunological state wherein a bias toward T-helper type 2 (Th2) dominance exists. When the Th1/Th2 balance is disrupted, the mother becomes vulnerable to viral infections, which are more effectively contained by Th1 cells. Thus, an integrated approach is required if these unique challenges occur in pregnancies affected with SARS-CoV-2 infection [8]. Although the risk of severe COVID-19 in pregnancy appears to be no greater than that in the general population, pregnant women are still highly at risk for viral respiratory infections and severe pneumonia because of the unique physiological changes in their immune and cardiopulmonary systems [9]. Most pregnant people have mild to moderate symptoms only; however, SARS-CoV-2 infection is found to be more severe in pregnant people than in their nonpregnant counterparts, with an increased risk of hospital admission, intensive care unit stay, and even death [10].

A randomized controlled trial (RCT) demonstrated that remdesivir administration was safe in pregnant patients with Ebola, without significant adverse effects [11]. Moreover, the use of remdesivir for moderate-to-severe COVID-19 who need oxygenation treatment was demonstrated to have a modest benefit [12]. However, its use in pregnant people with SARS-CoV-2 infection is still currently poorly investigated. In addition, viral infection therapy is challenging for clinicians because of the elusive biological behavior of viruses. Therefore, to facilitate elucidating the antiviral therapy in pregnancy, we conducted a systematic review to critically evaluate and summarize the latest evidence of remdesivir for COVID-19 in terms of its efficacy and safety profile among pregnant women.

## 2 Methods

This systematic review conformed to the guidelines of Preferred Reporting Items for Systematic Review and Meta-Analysis (PRISMA) 2020 [13] and has been registered at PROSPERO database (CRD42021262700).

### 2.1 Eligibility criteria

This review included the following study types: retrospective, prospective, cohort, RCT, case control, cross sectional, crossover, case series, and case reports. Studies were selected according to the following criteria: (1) adult pregnant population (≥18 years old); (2) remdesivir as the study of interest; (3) eligible studies reporting at least one of our outcomes of interest; and (4) English language. Our primary outcomes included clinical recovery, hospital discharge, and adverse events. Neonatal outcomes and laboratory outcomes constituted our secondary outcomes. Conversely, we excluded review articles, nonhuman studies, irrelevant articles, and duplications.

### 2.2 Search strategy and selection of studies

**Figure 1:**
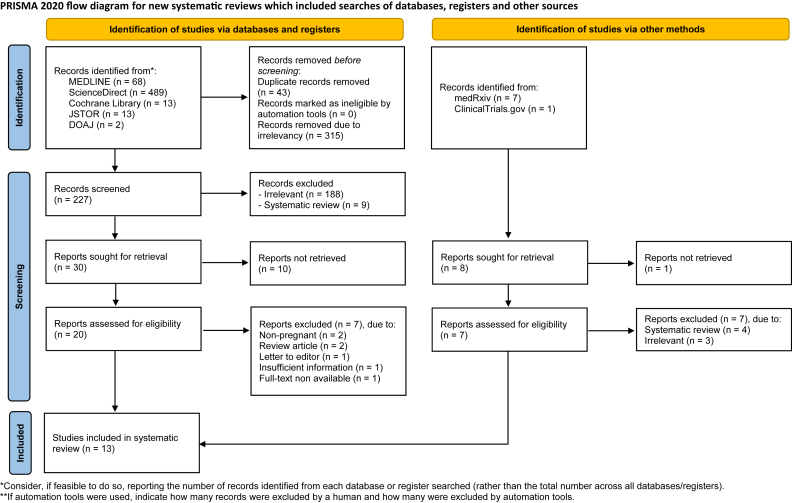
PRISMA flow diagram for included studies.

On July 26, 2021, articles published in trial registries (ClinicalTrials.gov, the WHO Clinical Trial Registry, and the EU Clinical Trial Registry) and databases (MEDLINE, ScienceDirect, Cochrane Library, Journal Storage [JSTOR], and Directory of Open Access Journals [DOAJ]) were thoroughly searched using the following keywords: “(COVID) OR (SARS-COV-2)) AND ((Pregnant) OR (Pregnancy) OR (Obstetric)) AND (Remdesivir).” Manual search, including in bioRxiv and medRxiv, and bibliographical search were also conducted to obtain additional evidence. Detailed search strategies are available in Supplementary Materials. All studies retrieved from the electronic searches were then exported into Mendeley reference manager for duplication removal and screening. To identify potentially eligible studies, two review authors (NRP and IAW) individually screened the titles and abstracts of the articles and subsequently screened the full texts independently. Any disagreements between them were resolved by discussion until consensus was reached. Excluded studies were described in the PRISMA flow diagram alongside their reasons for exclusion (Figure 1).

### 2.3 Data extraction

Using the structured and standardized forms from each selected study, the review authors independently extracted relevant data. They extracted the following information: first authors’ names and publication year, study design, country of origin, sample size, patient age, gestational age, disease severity, comorbidities, remdesivir dosage and duration, concurrent therapy, and outcomes (clinical outcome, hospital discharge, laboratory outcome, adverse event, pregnancy, and neonatal outcome). Any disagreements were discussed by these authors until consensus was reached.

### 2.4 Quality assessment

The risk of bias from each included study was independently assessed by two review authors (DSB and IAW) using the Newcastle–Ottawa Scale (NOS) assessment tool for cohort studies and Joanna Briggs Institute (JBI) critical appraisal checklist for case report and case series studies [14, 15]. The NOS contains eight items within three domains (patient selection, comparability, and outcomes). A study with scores of 7–9, 4–6, and 0–3 was considered to be high, moderate, and low in quality, respectively. Any discrepancies were resolved by discussion until consensus was reached.

### 2.5 Statistical analysis

Considering the important differences in the comparison of each study and various outcome measures, we could not generate meta-analyses of the included studies; rather, we narratively synthesized the evidence.

## 3 Results

### 3.1 Study selection

Database and manual searching yielded 585 and 8 records, respectively. After title and abstract screening, 27 potentially eligible articles were selected for review, and after full-text assessment, 13 studies were included for narrative synthesis. The study selection process is summarized in the PRISMA flow diagram alongside the reason for exclusion (Figure 1).

### 3.2 Quality assessment

Two cohort studies were considered to be high in quality according to NOS (S2 Table). The quality assessment of case reports and case series using the JBI critical appraisal checklist is summarized in Supplementary Materials (S3–S4 Table).

### 3.3 Study characteristics

Ultimately, this review included 13 observational studies with 113 pregnant people who received remdesivir treatment. Most of these observational studies (2 cohort studies, 4 case series, and 4 case reports) were conducted in the United States [16, 17, 18, 19, 20, 21, 22, 23, 25, 28]. Other case reports were conducted in Indonesia [24], India [26], and Japan [27].

### 3.4 Patient characteristics

The median (interquartile range [IQR]) patient age was 33 (21–43) years. Regarding disease severity, 78% of the patients were severe to critical, and 22% were moderate. Seven out of 13 studies reported comorbid data in participants, whereas the remaining studies only reported disease severity. Among the comorbidities were obesity (16%), asthma (13%), gestational diabetes (10%), and hypertension (9%) [16]. The gestational age of the participants were mostly 26 weeks or more, except in two studies wherein 22 weeks was reported [18, 20].

### 3.5 Clinical outcomes

All 13 observational studies reported clinical outcomes [16, 17, 18, 19, 20, 21, 22, 23, 24, 25, 26, 27, 28]. Cohort studies that compared oxygen needs between pregnant and postpartum women used a modified ordinal scale with the following criteria: (6) death; (5) hospitalized, requiring mechanical ventilation (intermittent mandatory ventilation) and/or extracorporeal membrane oxygenation; (4) hospitalized, requiring high-flow oxygen therapy and/or noninvasive positive pressure ventilation; (3) hospitalized, requiring supplemental low-flow oxygen therapy; (2) hospitalized on room air; (1) discharge. A 2-point improvement or any (1-point) improvement of the ordinal scale defined recovery. After 28 days, 93% of pregnant women and 89% of postpartum women had recovered. The highest rates of clinical recovery among pregnant women not requiring invasive ventilation were as follows: 98% achieving any clinical improvement, 89% postpartum having 2-point improvement from baseline, and 95% were discharged [16]. The median time to recovery was 5 days in pregnant women who did not receive invasive ventilation, and 13 days in those who received (*P* < 0.001). Pregnant and postpartum women who were invasively ventilated at baseline had similar times to extubation (11 vs. 7 days, *P* = 0.61). Multivariate analysis revealed a significantly longer recovery time in pregnant women who already received mechanical ventilation at baseline (hazard ratio, 0.34 [95% confidence interval, .20–.59]; *P* = 0.0001) [16].

A cohort study evaluated clinical recovery based on remdesivir administration time after hospital admission [17]. Clinical recovery reached 100% (17/17) at hospitalization day 7 among patients who received remdesivir within 48 h from admission. In contrast, all patients (7/7) who started remdesivir 48 h after admission failed to achieve clinical recovery. Only 27.3% (3/11) of patients recovered after being treated merely with antibiotics with or without glucocorticoids (*P* ˂ 0.001). In patients with moderate COVID-19 symptoms at the start of treatment, remdesivir was superior in achieving clinical recovery on hospitalization day 7 (15/15) compared with antibiotics with or without glucocorticoids (3/11) (*P* < 0.001) [17].

Based on consecutive case series and case reports, clinical recovery from oxygen-need reduction was 90% (20/22), and the average hospitalization duration was 13.3 ± 11 days [18, 19, 20, 21, 22, 23, 24, 25, 26, 27, 28]. In the case series of five pregnant people with moderate to severe COVID-19, clinical conditions during hospitalization improved rapidly following remdesivir treatment, and the hospitalization time was shorter [24]. However, one case report of a 42-year-old pregnant patient (with positive COVID-19 RT-PCR, 78% oxygen saturation without supplemental oxygen, and 50–60 breaths per minute [respiratory rate]) received the following treatment: 20 mg of intravenous dexamethasone for 5 days and then 10 mg for 5 days; 200 mg of remdesivir as a loading dose and then 100 mg every 24 h for 9 days; convalescent plasma on hospital day 2; and azithromycin plus ceftriaxone for empirical treatment of possible superimposed bacterial pneumonia. On hospital day 11, >95% oxygen saturation could not be achieved despite maximum oxygen supplementation. Nevertheless, the patient was finally discharged on day 52 with continued nursing care and home oxygen treatment [21]. In a case report by Schnetter et al., patients who experienced rapid clinical decompensation and developed severe COVID-19-related ARDS had clinically improved despite synchronized intermittent mandatory ventilation with 35% fraction of inspired oxygen. They also performed spontaneous breathing trials on hospitalization day 17 [28].

### 3.6 Laboratory outcomes

Ten observational studies reported laboratory outcomes [16, 17, 18, 19, 20, 22, 24, 25, 26, 27]. In cohort studies, 67% of participants had treatment-emergent–graded laboratory abnormalities based on the criteria of “The Division of AIDS Table for Grading the Severity of Adult and Pediatric Adverse Event version 2.1.” Aspartate aminotransferase (AST) and alanine aminotransferase (ALT) elevations were mostly grade 1 (1.25 < 2.5 × ULN) and grade 2 (2.5 to <5.0 × ULN). Grade 3 elevations (>5 × ULN) of ALT and AST occurred in 9% (6/64) and 5% (3/62) of pregnant participants, respectively. However, no grade 4 (>10 × ULN) ALT or AST elevation was reported [16].

### 3.7 Pregnancy and neonatal outcomes

Eleven observational studies investigated pregnancy and neonatal outcomes [16, 17, 19, 21, 22, 23, 24, 25, 26, 27, 28]. In cohort studies, 26 births have been reported wherein 27% (7/26) and 73% (19/26) were through vaginal delivery and cesarean section, respectively. Of 19 cesarean births, 17 were of emergency. In addition, 69% (18/26) of the deliveries were often very preterm (24–32 gestational weeks) [16]. Incidental oligohydramnios that occurred within 5 days after remdesivir treatment completion was found in 12.5% (3/24). Four of 27 (15%) infants had growth restriction (1st–7th percentile), with no major histopathologic abnormalities in their placentas [17]. Additionally, a 32-year-old woman at 17 gestational weeks experienced spontaneous miscarriage [16]. In case report and case series studies, 71% (12/17) and 29% (5/17) births were through cesarean section and vaginal delivery, respectively [19, 21, 22, 23, 24, 25, 26, 27, 28]. Among these births, no vertical transmission of COVID-19 occurred [16, 17, 19, 21, 22, 23, 24, 25, 26, 27, 28]. Four of 17 births (23.5%) were premature [21, 26, 27, 28]; while the others were successfully stabilized [19, 22, 23, 24, 25], one neonate required neonatal intensive care unit (NICU) admission [21].

### 3.8 Adverse events

Seven observational studies reported adverse events following remdesivir treatment [16, 17, 19, 20, 22, 23, 26]. The total incidence of any adverse events in remdesivir for 10 days was 38.5% (30/78) [16,[19], [20], [21],^23^,^26^,^28^], and that for 5 days was 29% (9/31) [17,22,24,25]. No maternal death occurred [16, 17, 18, 19, 20, 21, 22, 23, 24, 25, 26, 27, 28]. Among pregnant participants, 18% (12/67) experienced serious adverse events, including fetal death (2%, 1/67), cardiac arrest (2%, 1/67), and acute respiratory distress syndrome (2%, 1/67), and 33% (22/67) experienced mild adverse events [16]. Meanwhile, the most reported adverse event following remdesivir treatment was transaminitis (33%, 8/24) [17]. In other studies, transaminitis was also the most common adverse event (45%, 10/22) [19,20,22,23,26]. Remdesivir treatment was discontinued in two patients after developing severe transaminitis [22, 23].

## 4 Discussion

Positive results from early studies among the general population attracted the attention of the remdesivir administration in pregnant women with COVID-19. In this study, nine case reports and case series demonstrated clinical recovery after remdesivir treatments [18, 19, 20, 22, 23, 24, 25, 26, 27]. The clinical recovery rate was significantly higher in patients who received remdesivir within 48 h from admission than those beyond 48 h or those treated only with antibiotics with or without glucocorticoids [17]. Intriguingly, a case report of pregnancy with critical respiratory failure related to COVID-19 reported that the patients did not clinically improve after receiving remdesivir and other treatments; rather, they clinically recovered after delivery [21]. Regarding those pregnant patients who continued to deliver, cesarean section was more frequent than vaginal delivery. Most cesarean deliveries were performed for emergency reasons. Most of the deliveries were very preterm (24–32 gestational weeks) despite no obstetric indication for preterm delivery, such as spontaneous preterm labor, preeclampsia, and placental abruption [16].

Four out of 17 births were preterm; however, all neonates were reported in stable condition, except for one who required NICU admission because of adrenal insufficiency due to maternal dexamethasone treatment [21, 26, 27, 28]. Moreover, a cohort reported incidental oligohydramnios in three patients without risk factors such as hypertensive disease, tobacco use, and fetal growth restriction. Oligohydramnios is idiopathic, whether it is attributed to remdesivir or COVID-19 remains unclear [17]. Additionally, a patient with a history of intravenous drug use experienced a spontaneous miscarriage at 17 gestational weeks. This patient was found to have concurrent Staphylococcus aureus bacteremia, tricuspid valve endocarditis, and septic arthritis [16]. No cases of vertical transmission and significant histopathologic abnormalities in the placentas were reported [17].

The most common adverse event following remdesivir treatment was transaminitis [16, 17, 19, 20, 22, 23, 26], where most events were categorized as mild according to the criteria of “The Division of AIDS Table for Grading the Severity of Adult and Pediatric Adverse Event, version 2.1” [16]. However, remdesivir was discontinued in two patients because of worsening transaminitis [22, 23]. One was related to cholestasis [22], while the other was unclear [23]. Therefore, despite the treatment with remdesivir, COVID-19 was suspected to be the cause, considering that these two patients experienced transaminitis even before remdesivir treatment was started [23, 26]. In addition, the duration of remdesivir administration may be associated with the number of adverse events [16, 17].

Previously published RCT study by Beigel et al. revealed that remdesivir could hasten the recovery of adults hospitalized with COVID-19 and reduce the risk of secondary lower respiratory tract infection, especially in those who require low-flow oxygenation [29]. Therefore, on September 1, 2020, the National Institute of Health guidelines recommended prioritizing the use of remdesivir for all hospitalized patients with COVID-19 who require low-flow oxygen supplementation [30]. In contrast, prior systematic review by Ansems et al. summarized that the effects of remdesivir in hospitalized adults showed little or no difference to all-cause mortality at up to day 28, the duration to liberation from invasive mechanical ventilation, and the risk of clinical worsening in terms of the new need for mechanical ventilation at up to day 28 [31].

Among pregnant women with SARS-CoV-2 infection, the administration of 5-day remdesivir could clinically improve moderate COVID-19 [17], consistent with the finding in the general population [32]. Furthermore, Burwick et al. also reported that 10-day remdesivir administration could clinically improve severe COVID-19 in pregnant and postpartum patients [16]. On the other hand, spinner et al. study revealed that the addition of remdesivir treatment duration among the general population with moderate COVID-19 did not present significant clinical improvement compared with the standard care [32]. The two abovementioned RCTs by Beigel et al. and Spinner et al. investigated the use of remdesivir as an addition to standard care, including corticosteroids [29, 32]. Moreover, our included studies widely used remdesivir, as an addition to other treatments, and the clinical condition were varied from moderate to critical. Meanwhile, drugs such as corticosteroids and tocilizumab were associated with clinical improvement among moderate to critical COVID-19 patients [33, 34, 35], remdesivir monotherapy could already provide clinical improvement for patients with moderate COVID-19, as reported by a cohort study conducted by Nasrallah et al. [17]. This finding may strengthen the fact that remdesivir improves the clinical outcomes of patients with COVID-19. Nevertheless, more studies are needed to confirm the benefit of remdesivir monotherapy; currently, no result from RCT phase 3 is available [36].

The benefits and side effects of remdesivir treatment in the first trimester of pregnancy remained unknown as all studies included in this systematic review only included pregnant women in the second and third trimesters. Given the lack of studies evaluating the efficacy and safety of remdesivir administered during the first trimester, careful consideration is required when initiating this treatment in this period. In comparison, remdesivir is a safe treatment option for Ebola virus infection in pregnancy, with no significant safety concern on the fetus and the mother [11]. This drug was also used for treating congenital Ebola infection, with no evidence for drug toxicity [37]. In the general population with COVID-19, the most common side effects following remdesivir treatment were nausea, transaminitis, and respiratory failure [38]. This finding is consistent with the present study's result wherein transaminitis was the most commonly experienced adverse reaction [16, 17]. In addition, ALT levels should be monitored serially for pregnant people who are on remdesivir treatment. Remdesivir treatment may need to be stopped when the ALT level rises to more than ten times the normal upper limits or if signs and symptoms of liver inflammation are noted [39]. Among the general population, 5-day treatment of remdesivir for COVID-19 showed fewer adverse events than 10-day treatment [38]. In the present study, pregnant people who received 5-day remdesivir treatment also displayed fewer adverse events, which were not even serious. However, the clinical outcomes of remdesivir in terms of its efficacy remain unclear. Therefore, the length of its use should be based on the clinical condition of the patients. Pregnant women who still require mechanical ventilation after five days of remdesivir treatment may continue until ten days with monitoring ALT levels and adverse reactions carefully.

Our systematic review included a comprehensive literature search with specific criteria for inclusion and quality appraisal. To our knowledge, it is the first to summarize the evidence of remdesivir for COVID-19 treatment in the pregnant population. Although the findings were limited by the quality and scope of data in the reports that were not uniform, we incorporated extensive efforts to obtain the shreds of evidence from eligible studies dominated by case series and case reports. However, the type of evidence reviewed (case series and reports) was uncontrolled and remained at the bottom of the hierarchy of evidence. Thus, the inferences are inherently inadequate. Nevertheless, we have ensured that our systematic review methods have been transparently reported to facilitate future updating within this discussion area. Future RCTs are required to provide compelling evidence regarding the efficacy and safety of remdesivir administration among the pregnant population.

## 5 Conclusion

The efficacy and safety profile of remdesivir among pregnant women with COVID-19 remain inconclusive. Despite the fact that better clinical status at baseline with earlier remdesivir treatment may result in better clinical outcomes, careful monitoring of adverse reactions and transaminase enzyme levels should be carefully monitored. Furthermore, considering that all findings in this study were based on observational studies, further studies with careful adjustment of confounders are required as well as RCTs, thus allowing us to draw more reliable conclusions on the potential benefits and harms of remdesivir.

## Declarations

### Author contribution statement

David Setyo Budi, Nando Reza Pratam, Ifan Ali Wafa and Citrawati Dyah Kencono Wungu: Conceived and designed the experiments; Performed the experiments; Analyzed and interpreted the data; Wrote the paper.

Manesha Putra and Manggala Pasca Wardhana: Conceived and designed the experiments; Performed the experiments; Contributed reagents, materials, analysis tools or data.

### Funding statement

This research did not receive any specific grant from funding agencies in the public, commercial, or not-for-profit sectors.

### Data availability statement

Data included in article/supplementary material/referenced in article.

### Declaration of interests statement

The authors declare no conflict of interest.

### Additional information

No additional information is available for this paper.

## Appendix A Supplementary data

Supplementary Materials Heliyon Supplementary Materials Heliyon

Prisma 2020 Checklist_Heliyon Prisma 2020 Checklist_Heliyon
