# Supplementary material for: Remdesivir for pregnancy: A systematic review of antiviral therapy for COVID-19
Source: Heliyon. 2022 Jan 31;8(1):e08835. doi: 10.1016/j.heliyon.2022.e08835 (PMC8802673; doi:10.1016/j.heliyon.2022.e08835)
Supplement: Supplementary Materials Heliyon [file mmc1.docx]

**SUPPLEMENTARY MATERIALS**

**S1.** **Literature Search**

- Keywords

#1 (Covid) OR (SARS-CoV-2)

#2 (Pregnant) OR (Pregnancy) OR (Obstetric)

#3 (Remdesivir)

- Search Results

| **Database** | **Keywords** | **Search Result** | **Search-time** |
| --- | --- | --- | --- |
| MEDLINE | #1 AND #2 AND #3 | 68 | July 26, 2021 |
| ScienceDirect | #1 AND #2 AND #3 | 489 | July 26, 2021 |
| Cochrane Library | #1 AND #2 AND #3 | 13 | July 26, 2021 |
| JSTOR | #1 AND #2 AND #3 | 13 | July 26, 2021 |
| DOAJ | #1 AND #2 AND #3 | 2 | July 26, 2021 |
| medRxiv | #1 AND #2 AND #3 | 7 | July 26, 2021 |
| Clinicaltrial.gov | #1 AND #2 AND #3 | 1 | July 26, 2021 |

**S2 Table. Newcastle-Ottawa Scale (NOS) quality assessment of each included cohort study**

| Study | **Selection** | | | | **Comparability** | | **Outcome** | | |  |
| --- | --- | --- | --- | --- | --- | --- | --- | --- | --- | --- |
|  | Representativeness of exposed cohort | Selection of nonexposed cohort | Ascertainment of exposure | Demonstration that outcome of interest was not present at start of study | Adjust for the most important risk factors | Adjust for other risk factors | Assessment of outcome | Follow-up length | Loss to follow-up rate | Total quality score |
| Burwick et al., 2020 | - | 🟊 | 🟊 | 🟊 | 🟊 | 🟊 | 🟊 | 🟊 | 🟊 | 8 |
| Nasrallah et al., 2021 | - | 🟊 | 🟊 | 🟊 | 🟊 | 🟊 | 🟊 | 🟊 | 🟊 | 8 |

**S3 Table. Joanna Briggs Institute (JBI) critical appraisal for case series study**

| No | Checklist questions | Igbinosa et al., 2020 | McCoy et al., 2020 | Saroyo et al., 2021 | Singh and Choudhary, 2021 |
| --- | --- | --- | --- | --- | --- |
| 1. | Were there clear criteria for inclusion in the case series? | Yes | Yes | Yes | Yes |
| 2. | Was the condition measured in a standard, reliable way for all participants included in the case series? | Yes | Yes | Yes | Yes |
| 3. | Were valid methods used for identification of the condition for all participants included in the case series? | No | Yes | Yes | Yes |
| 4. | Did the case series have consecutive inclusion of participants? | No | Yes | No | No |
| 5. | Did the case series have complete inclusion of participants? | No | Yes | No | No |
| 6. | Was there clear reporting of the demographics of the participants in the study? | Yes | Yes | Yes | Yes |
| 7. | Was there clear reporting of clinical information of the participants? | No | Yes | Yes | Yes |
| 8. | Were the outcomes or follow up results of cases clearly reported? | Yes | Yes | Yes | Yes |
| 9. | Was there clear reporting of the presenting site(s)/clinic(s) demographic information? | No | Yes | Yes | Yes |
| 10. | Was statistical analysis appropriate? | NA | NA | NA | NA |

**S4 Table. Joanna Briggs Institute (JBI) critical appraisal for case report study**

| No | Checklist questions | Naqvi et al., 2020 | Maldarelli et al., 2020 | Anderson et al., 2020 | Jacobson et al., 2020 | Dande et al., 2020 | Chinen et al., 2021 | Schnettler et al., 2020 |
| --- | --- | --- | --- | --- | --- | --- | --- | --- |
| 1. | Were patient’s demographic characteristics clearly described? | Yes | Yes | Yes | Yes | Yes | Yes | Yes |
| 2. | Was the patient’s history clearly described and presented as a timeline? | Yes | Yes | Yes | Yes | Yes | Yes | Yes |
| 3. | Was the current clinical condition of the patient on presentation clearly described? | Yes | Yes | Yes | Yes | Yes | Yes | Yes |
| 4. | Were diagnostic tests or assessment methods and the results clearly described? | Yes | Yes | Yes | Yes | Yes | Yes | Yes |
| 5. | Was the intervention(s) or treatment procedure(s) clearly described? | Yes | Yes | Yes | Yes | Yes | Yes | No |
| 6. | Was the post-intervention clinical condition clearly described? | Yes | Yes | Yes | Yes | Yes | Yes | No |
| 7. | Were adverse events (harms) or unanticipated events identified and described? | Yes | Yes | Yes | No | Yes | No | No |
| 8. | Does the case report provide takeaway lessons? | Yes | Yes | Yes | Yes | Yes | Yes | Yes |
